# The Baby Steps Web Program for the Well-Being of New Parents: Randomized Controlled Trial

**DOI:** 10.2196/23659

**Published:** 2021-11-26

**Authors:** David John Kavanagh, Jennifer Connolly, Jane Fisher, W Kim Halford, Kyra Hamilton, Leanne Hides, Jeannette Milgrom, Heather Rowe, Paul A Scuffham, Katherine M White, Anja Wittkowski, Shelley Appleton, Davina Sanders

**Affiliations:** 1 Centre for Children’s Health Research Queensland University of Technology South Brisbane Qld Australia; 2 School of Psychology and Counselling Queensland University of Technology South Brisbane Qld Australia; 3 School of Public Health and Preventive Medicine Monash University Melbourne Australia; 4 School of Psychology The University of Queensland Brisbane Australia; 5 School of Applied Psychology Griffith University Brisbane Australia; 6 Menzies Health Institute Queensland Griffith University Brisbane Australia; 7 Perinatal and Infant Research Institute Austin Health Melbourne Australia; 8 Melbourne School of Psychological Sciences University of Melbourne Melbourne Australia; 9 School of Health Sciences University of Manchester Manchester United Kingdom

**Keywords:** perinatal, depression, prevention, men, self-guided, internet

## Abstract

**Background:**

New parents face increased risks of emotional distress and relationship dissatisfaction. Digital interventions increase support access, but few preventive programs are optimized for both parents.

**Objective:**

This study aims to conduct the first randomized controlled trial on universal self-guided digital programs to support positive perinatal adjustment of both mothers and fathers. Effects of childcare information (*Baby Care*) and information plus an interactive program (*Baby Steps Wellbeing*) were compared from the third trimester baseline to 3 and 6 months subsequently.

**Methods:**

The study recruited 388 co-parenting male-female adult couples expecting their first single child (26-38 weeks’ gestation), using web-based registration. Most (337/388, 86.8%) were obtained from prenatal hospital classes. Couples’ randomization was automated and stratified by Edinburgh Postnatal Depression Scale (EPDS) scores (50% couples scored *high* if either mother >7, father >5). All assessments were web-based self-reports: the EPDS and psychosocial quality of life were primary outcomes; relationship satisfaction, social support, and self-efficacy for parenting and support provision were secondary. Linear mixed models provided intention-to-treat analyses, with linear and quadratic effects for time and random intercepts for participants and couples.

**Results:**

Selection criteria were met by 63.9% (248/388) of couples, who were all randomized. Most participants were married (400/496, 80.6%), tertiary educated (324/496, 65.3%), employed full time (407/496, 82%), and born in Australia (337/496, 67.9%). Their mean age was 32.2 years, and average gestation was 30.8 weeks. Using an EPDS cutoff score of 13, 6.9% (18/248) of men, and 16.1% (40/248) of women screened positive for depression at some time during the 6 months. Retention of both partners was 80.6% (201/248) at the 6-month assessments, and satisfaction with both programs was strong (92% ≥50). Only 37.3% (185/496) of participants accessed their program more than once, with higher rates for mothers (133/248, 53.6%) than fathers (52/248, 20.9%; *P<*.001). The EPDS, quality of life, and social support did not show differential improvements between programs, but *Baby Steps Wellbeing* gave a greater linear increase in self-efficacy for support provision (*P=*.01; Cohen *d*=0.26) and lower reduction in relationship satisfaction (*P=*.03; Cohen *d*=0.20) than *Baby Care* alone. Mothers had greater linear benefits in parenting self-efficacy over time than fathers after receiving *Baby Steps Wellbeing* rather than *Baby Care* (*P=*.01; Cohen *d*=0.51)*.* However, the inclusion of program type in analyses on parenting self-efficacy and relationship satisfaction did not improve model fit above analyses with only parent gender and time.

**Conclusions:**

Three secondary outcomes showed differential benefits from *Baby Steps Wellbeing*, but for one (parenting self-efficacy), the effect only occurred for mothers, perhaps reflecting their greater program use. Increased engagement will be needed for more definitive testing of the potential benefits of *Baby Steps* *Wellbeing* for perinatal adjustment.

**Trial Registration:**

Australian New Zealand Clinical Trials Registry ACTRN12614001256662; https://www.anzctr.org.au/Trial/Registration/TrialReview.aspx?id=367277

## Introduction

### Background

Psychological distress before and after childbirth is common in both parents. Although recent meta-analyses estimate that 12%-17% of recent mothers become depressed [1,2], the rate for fathers is approximately 8% [3]. Depression in one parent poses significant coping challenges for their partner and increases their own risk of depression [4-6]. However, few fathers receive treatment for depression or subclinical distress [7], and research on its treatment is sparse [8,9]. Preventive interventions targeting both parents’ distress and promoting quality of life are also likely to benefit the couple’s relationship [10,11] as well as their child’s emotional and social adjustment [12,13].

Digital delivery may be expected to reduce some of the stigma associated with help seeking, which is especially evident in fathers [7], and allow large-scale access to interventions aimed at preventing or treating distress. Web-based cognitive behavior therapy programs for postpartum depression have demonstrated a moderate average effect size in mothers (Cohen *d*=0.54) [14]. However, a search of published papers identified no research on interactive programs specifically for fathers, although interactive exercises for couples are sometimes included [15]. Even web-based information focusing on new fathers is limited [16,17].

*Baby StepsWellbeing* is a free, self-guided web program that is optimized for multiple devices and aims to prevent distress and promote the well-being of new parents [18]. It was developed by clinical psychologists and a midwife, with a particular focus on new fathers, and was based on unpublished qualitative research on the issues faced by 10 couples with babies aged 3-6 months and on semistructured interviews with 21 recent parents who reviewed the draft program. To maximize the engagement of new parents, the program contained 4 modules with practical tips on preparing for the baby and addressing issues related to feeding, sleep, and soothing (*Baby Care*). The full *Baby Steps Wellbeing* program had an additional four modules providing information and interactive problem solving on self-care, relationships, interacting with the baby and adjusting to the new roles, as well as a module specifically focused on fathers that covered the ways in which they could support their partner and share childcare tasks. Selecting a tip in *Baby Steps Wellbeing* allowed users to choose specific baby care and well-being tips and set calendar reminders to try them. They could also upload photos about good times with their baby for use at times of stress.

Digital provision of a support program does not guarantee that men will access it, and users of digital mental health services remain predominantly women [19]. Encouraging partners to enroll in a program together may be one way to maximize fathers’ engagement. This strategy may also provide opportunities to build mutual support and relationship satisfaction, which are key predictors of perinatal well-being [20]. Accordingly, this study required that both partners participate, protecting their confidentiality through separate usernames and passwords.

### Objective

The aim of this randomized controlled trial is to compare the efficacy of the full, interactive *Baby Steps Wellbeing* program (containing all modules and planning tools) with that of *Baby Care* informational tips alone over a period of 6 months from the third trimester of pregnancy onward. The primary outcomes were self-rated depression and psychosocial quality of life, whereas the secondary outcomes included relationship satisfaction, parental self-efficacy, and social support. We predicted that the *Baby Steps Wellbeing* program would result in better outcomes at 3 and 6 months from baseline, compared with *Baby Care* information only. Although we did not have a directional hypothesis about the relative effects of the programs on fathers and mothers, we tested whether the inclusion of an interaction of treatment and parent gender added to the prediction of outcomes. Effects for time were partialed into linear and quadratic effects because of the potential for deterioration in mental health and well-being during the early postpartum period, with some recovery at 6 months. We were interested both in whether the two treatments had differential effects linearly over the study period and whether they modified any tendency for short-term changes at around 2 weeks after childbirth (ie, 3 months from baseline).

## Methods

### Brief Description

A detailed description of the methodology is provided in the research protocol [21]. A summary is provided below, highlighting the changes from the published protocol.

### Ethical Approval, Inclusion Criteria, and Recruitment

The trial received ethical approval from the human research ethics committees of Queensland University of Technology (#1400000687) and Mater Health Services (HREC/14/MHS/166). The participants were co-parenting male-female couples aged 18 years or older who were expecting a single first child at 26-38 weeks’ gestation. Recruitment was timed during late pregnancy to engage parents before they faced the heavy demands of childcare [22], while ensuring that their entry was sufficiently proximal to the birth to maximize engagement and later recall. The participants were proficient in English, had access to the internet and a mobile phone, and had completed the baseline assessments. Recruitment occurred between March 2015 and October 2015, and the registration was conducted on the web. The study had a recruitment target of 240 couples, which was expected to enable the detection of a small effect size of f=0.082 (*α*=.05; power=0.80; repeated measures *r*=0.50, using repeated measures analysis of variance, G*Power 3.1 [Heinrich Heine University] [23]). However, we recruited 248 couples (124 couples in each arm). Most recruited couples (216/248, 87.1%) entered the study through prenatal parent education classes at the Mater Mothers’ Hospital, Brisbane, Australia, where a member of the research team presented information about the research trial immediately before the class. The remaining participants were recruited through a trade exhibit at the *Pregnancy, Babies, and Children’s Expo* in Brisbane, Australia (25/248, 10.1%); were referred by a friend, family member, or medical practitioner (6/248, 2.4%); or responded to advertising on a parenting website (1/248, 0.4%).

### Measures

#### Demographic and Pregnancy Data

At baseline, each parent reported their age, marital and employment status, education level, country of birth, years living in Australia, and indigenous background (where applicable). The assessment at 3 months after childbirth included weeks of gestation at birth, birth type, baby’s sex, and information about any inpatient care of the baby.

#### Program Use and Satisfaction

The number of log-ins, modules viewed, the number of saved action plans from baseline to 6 months, and the period between the first and last log-in to the program were downloaded from the program’s database. Overall program satisfaction and the program’s perceived relevance, usefulness, and ease of finding what the participants wanted were self-reported at 3 months on a scale from 0 (not at all) to 100 (extremely), and average scores across these four items are reported below (in this sample, Cronbach *α*=.93; corrected interitem correlations=0.73-0.91).

#### Outcomes

Except for the Assessment of Quality of Life-8 Dimensions (AQoL-8D), all self-reported outcomes used unweighted sums or averages of items. The primary outcome measures were the scores derived from the 10-item Edinburgh Postnatal Depression Scale (EPDS) [24,25], which provides a total score of 0-30, with higher scores indicative of depressive symptomatology, and the Psychosocial Super Dimension Scale from the AQoL-8D [26,27], which gives an average weighted score of increasing utility from 0 to 1.

Relationship satisfaction was measured using the 16-item Couples Satisfaction Index (CSI-16) [28], which gives a score of increasing satisfaction from 0 to 81. Social support was assessed using the 4-item version of the Medical Outcomes Study Social Support Survey (Short MOS-SSS) [29], which provides a total score from 4 to 20. A new measure of parenting self-efficacy used an average across the items on feeding, sleep, and settling (eg, “Thinking about the next 13 weeks, how confident are you with putting your baby to sleep?”), each of which was rated on an 11-point scale from 0 (not at all confident) to 100 (extremely confident). At baseline, the scale had high internal consistency (Cronbach *α*=.94; corrected item-total correlations=0.78-0.95). Self-efficacy for support provision to their partner used a single item (“Thinking about the next 13 weeks, how confident are you with providing support to your partner,” which was also rated on an 11-point scale from 0 to 100) that was moderately related to the CSI-16 (Spearman *ρ*=0.32; *P<*.001) and inversely related to the EPDS (Spearman *ρ*=–0.30; *P<*.001). Data on income, work time, productivity, and health care were also collected but will be reported in a separate paper.

### Web Programs

The participants randomized to the *Baby Care* trial arm received the four informational modules giving information and tips on getting prepared, feeding and soothing their baby, and improving their baby’s sleeping habits. A *Get Help* tab provided a list of relevant digital or telephone support services.

The participants receiving *Baby Steps Wellbeing* could also access modules on physical and emotional self-care, their relationship with their partner, changing roles, and interacting with their baby, as well as the module meant especially for fathers (ie, a total of 9 modules, including those focused on baby care). For either *Baby Care* tips or the ones in other modules, the participants in *Baby Steps Wellbeing* could identify goals, solve problems, develop a plan, set times to take action, and record their successful completion. A list of successfully completed plans was available. A web-based scrapbook was used to store photos of good times with their baby, and their dashboard presented due dates for their action plans, together with a rotating quiz question about baby care, a tip, and a scrapbook photo. The database for *Baby Steps Wellbeing* was hosted at the Queensland University of Technology. The participants were not given advice on the number of modules to access, and there was no limitation on the pace of module access. No changes to either program were made during the study, and no major technical issues were encountered.

All participants received automated text messages at 2, 4, 7, and 10 weeks after allocation, reminding them to log in to the program and select tips to apply. Texts sent to the *Baby Steps Wellbeing* participants also included a recommendation to review their goals and plans. A final SMS text message was sent to all participants, thanking them and expressing the hope that they found the website useful.

### Procedure

After the volunteers for the study registered on the web, they were emailed a link to the web-based consent form and eligibility screen. Prospective participants could also email or call the research team to ask questions about the trial. After informed consent and initial automated confirmation of eligibility, they were given a link to self-complete the baseline assessments. Both these and later measures were delivered through Qualtrics (Qualtrics, LLC; [30]) and stored separately from identifying data, using a numerical code for the couple plus a letter to signify father or mother. Completion of all items in each measure was required. If a respondent screened positive for a medium or high risk of major depression or self-harm (EPDS item 10 ≥1, or totals of ≥10 for mothers, ≥6 for fathers) at either the baseline or a later assessment, they were contacted by a member of the research team, who implemented a risk management plan [21].

The web-based completion of baseline measures by both the father and mother triggered a fully automated random allocation of the couple to a treatment by Goji, a web-based trial management system developed at Queensland University of Technology [31]. Randomization was performed in permuted blocks, stratified by EPDS (screening negative for anxiety or major or minor depression: mother ≤7 *and* father ≤5; screening positive: mother >7 *or* father >5) [25]. These cutoffs resulted in 123 couples (49.6%) screening positive—ie, approximating a median split of the couples on the EPDS.

At 13 and 26 weeks, the participants were sent automated emails with links to self-complete assessments on the web. All outcome and economic measures were readministered, together with details about the birth, a check on marital status, and ratings of program satisfaction. Nonresponse resulted in emails or calls by a research officer, who was blind to the treatment, to provide further reminders. The participants were given a retail store voucher for Aus $20 (approximately US $16) for each completed follow-up assessment.

### Statistical Analyses

The primary analyses adopted an intention-to-treat approach using RStudio version 1.3.1093 lme4 (RStudio, PBC), which provided mixed models analyses of data over the three measurement occasions (baseline, 3 months, and 6 months). Given the potential for correlations within couples, each analysis incorporated random intercepts for both the couple and individual participant. Preliminary analyses demonstrated that the inclusion of a first-order autocorrelation between test occasions did not improve predictions; therefore, it was not included in the reported analyses. As mothers are somewhat more vulnerable than fathers to issues with perinatal mental health and well-being [1-3], all analyses included effects for parent gender and the interaction of parent gender and time. In compliance with CONSORT (Consolidated Standards of Reporting Trials) guidelines, analyses of outcomes did not control for any differences between the treatments at baseline (contrary to the protocol paper) [21], but tests for any baseline differences are reported in the interests of full disclosure.

In the primary analyses, we compared two models: (1) a hypothesized model, examining the relative impact of the two programs, controlling for parent gender (ie, including effects of treatment×time, parent gender×time, and the relative main effects) and (2) the full factorial model, testing all potential effects. Post hoc analyses were also undertaken to examine a model that only included parent gender and time. The reported *t* tests used the Satterthwaite method, and the degrees of freedom were calculated using the Kenward-Roger method. The effect sizes in these analyses use Cohen *d*, with difference scores being divided by shared baseline SD units (taken from analysis of variance values of baseline scores, with treatment and parent gender as independent variables).

## Results

### Participant Characteristics

**Figure 1:**
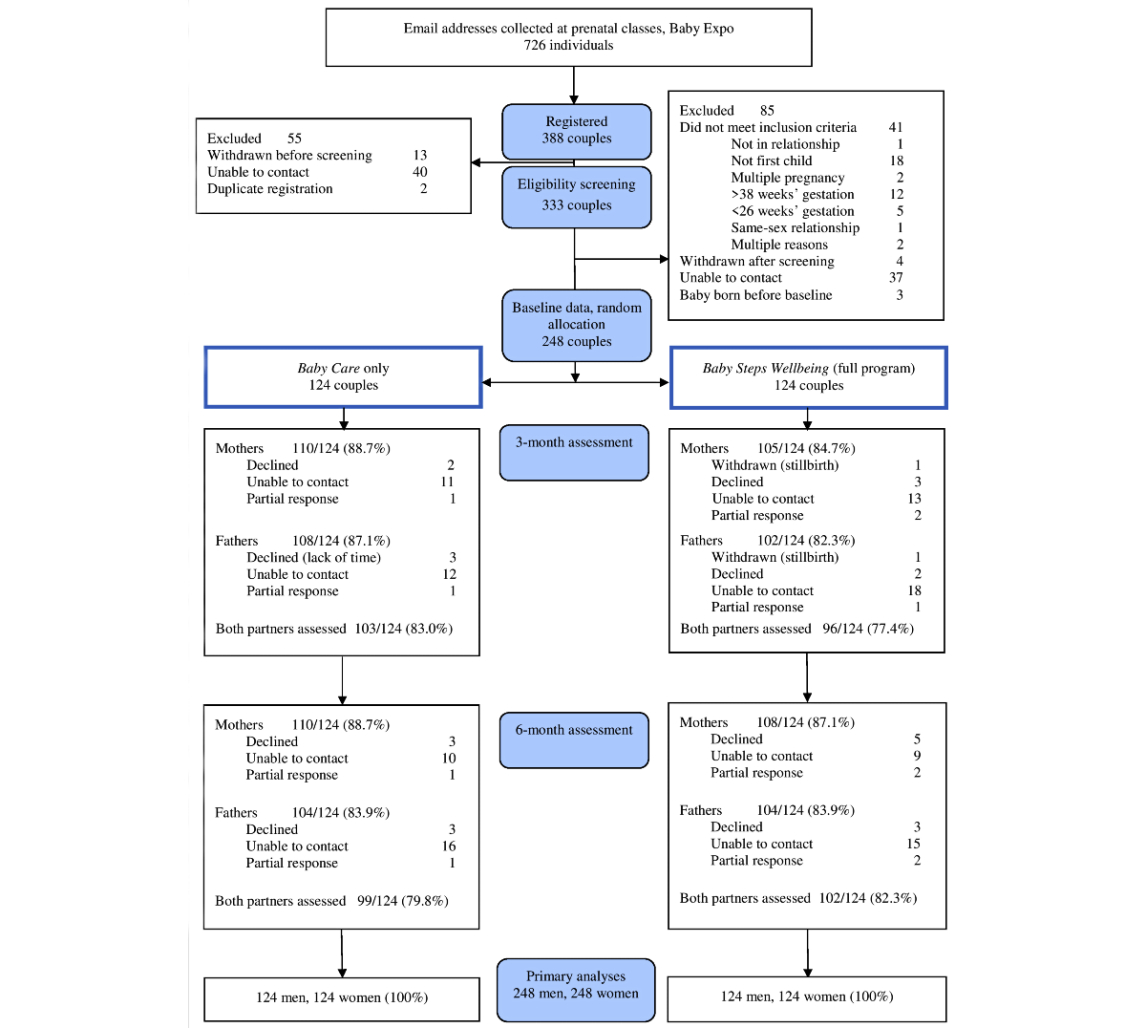
CONSORT (Consolidated Standards of Reporting Trials) diagram.

The CONSORT diagram is presented in Figure 1. Of the 726 people who expressed initial interest in the study, 388 couples registered; 248 (53.4) fulfilled the eligibility criteria and were randomly allocated to the two treatments. As shown in Figure 1, retention at 3- and 6-month assessments was high in both treatments. One couple had to be withdrawn from the study because of a stillbirth, but their baseline data were still included in the intention-to-treat analyses. An attempt was made to contact participants who screened positive for risk of depression or self-harm and implement the risk management procedure (112 at baseline, 175 at 3 months, 132 at 6 months), but no one had to be withdrawn from the study for this reason. Very few participants received concurrent mental health treatment during the study in either treatment (medication: 5%-7% and counseling: 4%-6%).

On average, the participants receiving *Baby Steps Wellbeing* entered the study a little less than a week earlier in the pregnancy compared with those receiving *Baby Care* (mean 30.4, SD 2.8 vs *Baby Care* mean 31.2, SD 3.1; *F*_1,246_=4.35; *P*=.04; η^2^=0.017), but there were no other significant differences between the treatments in terms of demographic characteristics. Tests of any differences between the fathers and mothers in the study are presented in Tables 1 and 2. Mothers were slightly younger than fathers on average and were more likely to have a university degree but less likely to be employed full time. At baseline, they had higher EPDS scores, lower AQoL-8D Psychosocial Super Dimension scores, and lower self-efficacy in the provision of social support. However, they had higher relationship satisfaction scores on the CSI-16 and higher perceived social support on the Short MOS-SSS.

**Table 1:** Baseline characteristics and scores by parent gender (fathers: n=248; mothers: n=248): categorical variables.

Variable			Fathers, n (%)		Mothers, n (%)		Chi-square test (*df*)			*P* value	
University degree			142 (57.2)		182 (73.3)		14.24 (1)			<.001	
**Employment**								13.98 (2)			.001
	Full time	219 (88.3)		188 (75.8)							
	Part time or casual	20 (8.0)		35 (14.1)							
	Unpaid leave, benefits or retired	9 (3.6)		25 (10.0)							
Indigenous			4 (1.6)		0 (0)		4.03 (1)			.045	
**Region of birth**								4.12 (2)			.13
	Australia	163 (65.7)		174 (70.2)							
	New Zealand or Pacific or United Kingdom or Ireland or North America or other European countries	53 (21.4)		36 (14.5)							
	Asia or Southeast Asia or Middle East or South America or Africa	32 (12.9)		38 (15.3)							

**Table 2:** Baseline characteristics and scores by parent gender (fathers: n=248; mothers: n=248): continuous variables.

Variable^a^	Fathers, predicted mean (SE)	Mothers, predicted mean (SE)	*t* test (*df*)	*P* value
Age (years)	33.0 (0.27)^b^	31.5 (0.27)^b^	15.77 (247.0)	<.001
Years living in Australia^c^	12.2 (1.02)^d^	10.5 (1.08)^e^	–1.37 (68.6)	.18
Depression (EPDS^f^ total)	4.47 (0.25)	5.42 (0.25)	2.81 (247.0)	.005
Quality of life (AQoL-8D^g^ Psychosocial)	0.474 (0.01)	0.434 (0.10)	–3.13 (247.0)	.002
Relationship satisfaction (CSI-16^h^)	69.8 (0.60)	71.9 (0.60)	3.26 (247.0)	.001
Social support (Short MOS-SSS^i^)	15.8 (0.21)	16.6 (0.21)	2.81 (247.0)	.005
Parenting self-efficacy	65.2 (1.20)	65.3 (1.20)	0.08 (247.0)	.94
Self-efficacy: support provision	81.2 (1.26)	71.8 (1.26)	–5.43 (247.0)	<.001

^a^Analyses of continuous variables include a random intercept for a couple.

^b^N=248.

^c^If born overseas.

^d^n=85.

^e^n=74.

^f^EPDS: Edinburgh Postnatal Depression Scale.

^g^AQoL-8D: Assessment of Quality of Life-8 Dimensions.

^h^CSI-16: Couples Satisfaction Index-16.

^i^MOS-SSS: Medical Outcomes Study Social Support Survey.

The participants had a median age of 32 (mean 32.2, SD 4.4; range 20-47) years, and 80.6% (400/496) were married. Most had a university degree (324/496, 65.3%), were employed full time (407/496, 82%), had been born in Australia (337/496, 67.9%), or had lived in Australia for a substantial period (mean 11.2 years, SD 9.5 years). Using a cutoff score of 12/13 on the EPDS [5] and no data substitution, 16.1% (40/248) of the mothers and 6.9% (18/248) of the fathers screened positive for depression at some time during the study. The rates at 3 months (approximately 2 weeks after childbirth) were 9.1% (20/217) for the mothers and 2.9% (6/209) for the fathers. If the Australian cutoffs for major or minor depression reported in the study by Matthey et al [25] were used (8/9 for mothers and 9/10 for fathers), 41.5% (103/248) of the mothers and 18.5% (46/248) of the fathers screened positive at some time, with the rates at 3 months being 27.2% (59/217) and 9.1% (19/209), respectively. The details are presented in Multimedia Appendix 1, Table S1.

### Program Satisfaction and Use

Satisfaction with the program was high among the participants who accessed it at least once (median 75, 92% ≥50; Table 3), with no substantial differences due to treatment or parent gender, but access and use were suboptimal, especially for the fathers. The median number of log-ins across the sample was 1 (range 0-23; 90th percentile=5). Only 36.7% (91/248) of *Baby Care* and 37.9% (94/248) of *Baby Steps Wellbeing* participants (*χ*^2^_1_=0.08; *P=*.781) accessed programs on two or more occasions. While 53.6% (133/248) of the mothers used a program at least twice, only 21.0% (52/248) of the fathers did so (*χ*^2^_1_=56.56; *P<*.001). Among the *Baby Steps Wellbeing* participants, 44.4% (55/124) of the mothers set a goal, whereas only 12.9% (16/124) of the fathers did so (*χ*^2^_1_=30.02; *P<*.001). These parent gender differences were observed for all continuous variables related to program use (Table 3; Multimedia Appendix 1, Table S2). Despite the fact that the fathers receiving *Baby Steps Wellbeing* could access more than twice the number of modules (nine) than those receiving *Baby Care* (four), fathers only accessed an average of just one module in either treatment. The significant interaction between treatment and parent gender (t_246_=2.35; *P*=.020) was a reflection of the fact that the mothers took greater advantage of the additional modules, although on average they still accessed fewer than half of those in *Baby Steps Wellbeing* (Table 2). More *Baby Care* modules were viewed by the participants in the *Baby Care* treatment (t_492_=–3.40; *P*<.001), but there were no statistically significant differences between the treatments in terms of log-ins or duration of use (Multimedia Appendix 1, Table S2). The baseline EPDS scores had a negligible association with the use of the programs (Multimedia Appendix 1, Table S2), but a weak negative correlation with program satisfaction (*r=*–0.18; *P*=.03).

**Table 3:** Program use and satisfaction^a^.

Variable		Values, predicted mean (SE)				
		Baby Care only			Baby Steps Wellbeing	
		Fathers	Mothers	Fathers		Mothers
Log-ins		1.09 (0.24)	2.67 (0.24)	0.89 (0.24)		2.77 (0.24)
Duration of use (days)		10.87 (4.08)	34.62 (4.08)	12.27 (4.08)		41.47 (4.08)
**Modules viewed**						
	Total	0.98 (0.17)	2.23 (0.17)	1.07 (0.17)		3.10 (0.17)
	Baby Care	0.98 (0.12)	2.23 (0.12)	0.40 (0.12)		1.56 (0.12)
Program satisfaction^b^		69.2 (2.03)	71.8 (2.01)	70.8 (2.10)		72.5 (2.06)

^a^Predicted means are derived from analyses that include a random intercept for a couple.

^b^Mean of responses across participants who had accessed a program to items on overall satisfaction, relevance, usefulness, and ease of finding what they wanted.

### Model Comparisons

Initially, a comparison was made of model fit from the hypothesized model and from a full factorial model. With the exception of parenting self-efficacy, the hypothesized model gave a better fit than the full factorial model in terms of both Akaike Information Criterion (AIC) and Bayesian Information Criterion (BIC), and the simpler model did not result in a significantly poorer likelihood ratio (Table 4). In the case of parenting self-efficacy, AIC favored the full factorial model. Although penalization for the greater number of predictors resulted in a slightly poorer BIC value, a better fit from the full model approached significance on the likelihood ratio test (*P*=.05). Accordingly, the subsequent main analyses on this variable used the full factorial model, whereas the hypothesized model was used for the remaining outcome variables. Results for each variable using the nonpreferred model are displayed in Multimedia Appendix 1, Table S3.

In additional post hoc analyses, we also tested the fit from a model with only parent gender and time to assess whether any significant effects for treatment resulted in an improved model fit overall (Multimedia Appendix 1, Table S4). For self-efficacy for support provision, this model gave a significantly poorer fit and a larger AIC value than the hypothesized model, although the reduction in model complexity resulted in a slightly lower BIC value. For the remaining outcomes, the AIC and BIC values were slightly smaller for the simpler model, but the likelihood ratio test was not significantly different. We note that these results for the model fit moderate the conclusions from the analyses on the effects of treatment on relationship satisfaction (CSI-16) and parenting self-efficacy, which are described below.

**Table 4:** Comparison of hypothesized and full factorial models for analyses of outcomes^a^.

Dependent variable				Parameters, N	AIC^b^	BIC^c^	Log likelihood	Deviance	Chi-square test (*df*=3)	*P* value
**Depression (EPDS^d^)**										
	Hypothesized model^e^			12	7247.7	7310.2	–3611.9	7223.7	3.34	.34
	Full model			15	7250.4	7328.5	–3610.2	7220.4	3.34	.34
**Quality of life (AQoL-8D^f^ Psychosocial)**										
	Hypothesized model^e^			12	–1361.4	–1298.9	692.7	–1385.4	4.90	.18
	Full model			15	–1360.3	–1282.2	695.2	–1390.3	4.90	.18
**Relationship satisfaction (CSI-16^g^)**										
		Hypothesized model^e^		12	9716.4	9778.9	–4846.2	9692.4	3.94	.27
		Full model		15	9728.5	9796.6	–4844.2	9688.5	3.94	.27
**Social support (Short MOS-SSS^h^)**										
		Hypothesized model^e^		12	6826.2	6888.7	–3401.1	6802.2	2.79	.42
		Full model		15	6829.4	6907.5	–3399.7	6799.4	2.79	.42
**Parenting self-efficacy**										
	Hypothesized model			12	11395	11458	–5685.7	11371	7.65	.05
	Full model^e^			15	11394	11472	–5681.9	11364	7.65	.05
**Self-efficacy for support provision**										
			Hypothesized model^e^	12	11560	11622	–5768.0	11536	4.03	.26
			Full model	15	11562	11640	–5765.9	11532	4.03	.26

^a^All models had random intercepts for the subject and couple. The hypothesized model included treatment, parent gender, time, treatment×time, and parent gender×time. The full model comprised the full factorial design.

^b^AIC: Akaike Information Criterion.

^c^BIC: Bayesian Information Criterion.

^d^EPDS: Edinburgh Postnatal Depression Scale.

^e^Preferred model.

^f^AQoL-8D: Assessment of Quality of Life-8 Dimensions.

^g^CSI-16: Couples Satisfaction Index-16.

^h^MOS-SSS: Medical Outcomes Study Social Support Survey.

### Primary Outcomes

**Figure 2:**
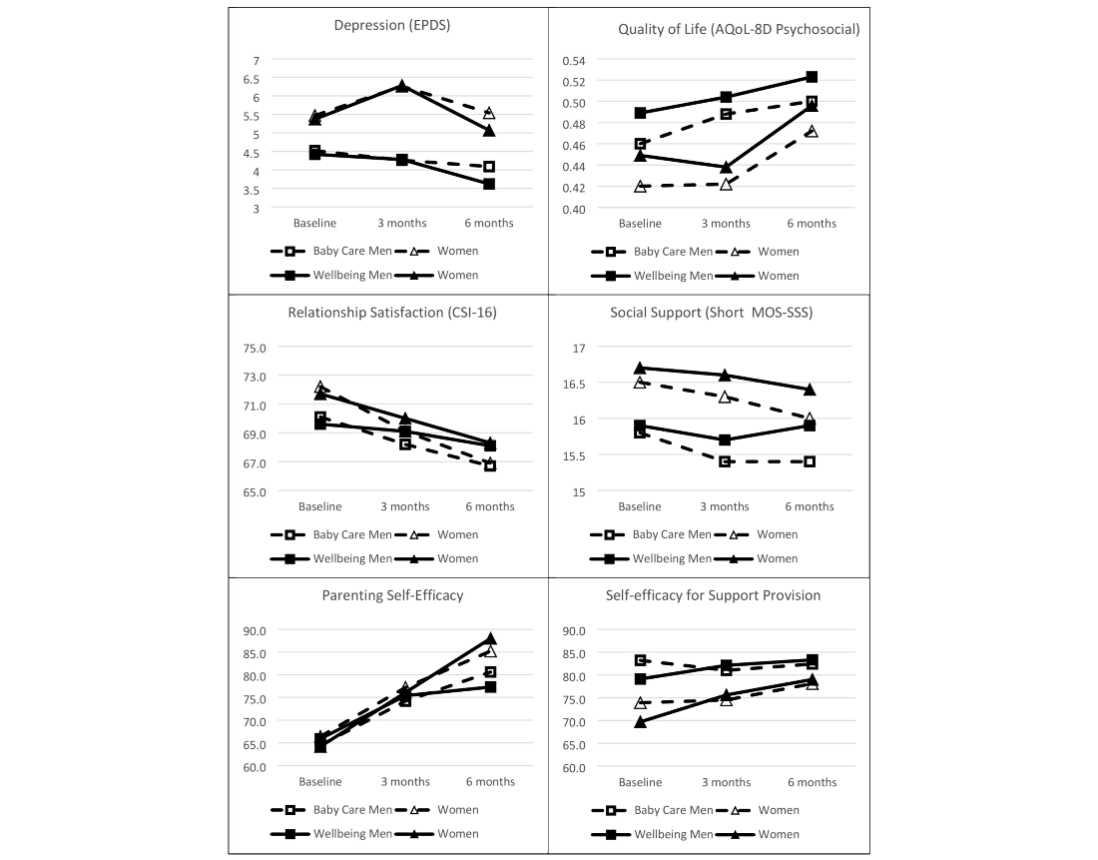
Predicted mean outcomes, using the preferred model. Estimations of means used the hypothesized model, except for parenting self-efficacy, which used the full factorial model. AQoL-8D: Assessment of Quality of Life-8 Dimensions; CSI-16: Couples Satisfaction Index-16; EPDS: Edinburgh Postnatal Depression Scale; MOS-SSS: Medical Outcomes Study Social Support Survey.

The effects for each outcome variable from the preferred model are displayed in Table 5, and the means are shown in Figure 2 and Multimedia Appendix 1, Table S5. Over the course of the study, the mothers had higher EPDS scores for depression (Cohen *d*=0.37) and a lower average quality of life on the AQoL-8D Psychosocial Super Dimension than the fathers (Cohen *d*=0.27). The EPDS scores did not significantly change across the sample from baseline to 6 months, but the mothers’ depression had a greater tendency to peak soon after childbirth, as shown by a significant parent gender×quadratric time effect (Table 5). For example, from baseline to 3 months, their EPDS scores rose 0.27 SD units more than those of the fathers. There was a linear improvement in quality of life over the study period (Cohen *d*=0.27), which once again was modified by an interaction between parent gender and quadratic time (eg, from baseline to 3 months): the psychosocial quality of life of the fathers increased by 0.16 SD units more than that of the mothers. However, there were no significant interactions between treatment and time on either primary outcome when the hypothesized model was used.

**Table 5:** Outcomes, using the preferred model.

Dependent variable			Estimate		SE		*t (df)*		*P* value
**Depression (EPDS^a^)**									
	Intercept	0.6744		0.2431		–2.77 (427.0)		.006	
	Treatment	–0.1820		0.2875		–0.63 (247.2)		.53	
	Parent gender	1.4675		0.2656		5.53 (246.6)		<.001	
	Linear time	–0.2993		0.2384		–1.26 (899.5)		.21	
	Quadratic time	0.0390		0.2417		0.16 (883.2)		.87	
	Parent gender×linear time	0.3537		0.2735		1.29 (896.2)		.196	
	Parent gender×quadratic time	–0.6512		0.2799		–2.33 (886.6)		.02	
	Treatment×linear time	–0.2681		0.2737		–0.98 (894.7)		.33	
	Treatment×quadratic time	–0.2556		0.2800		–0.91 (885.4)		.36	
**Psychosocial quality of life (AQoL-8D^b^ Psychosocial)**									
	Intercept	0.0142		0.0120		1.18 (381.2)		.24	
	Treatment	0.0225		0.0145		1.50 (247.7)		.13	
	Parent gender	–0.0445		0.0114		–3.89 (249.3)		<.001	
	Linear time	0.0282		0.0091		3.11 (888.9)		.002	
	Quadratic time	–0.0073		0.0092		–0.79 (876.9)		.43	
	Parent gender×linear time	0.0088		0.0104		0.85 (888.7)		.398	
	Parent gender×quadratic time	0.0265		0.0107		2.48 (880.2)		.01	
	Treatment×linear time	–0.0038		0.0104		–0.37 (884.3)		.71	
	Treatment×quadratic time	0.0090		0.0107		0.84 (877.1)		.40	
**Relationship satisfaction (CSI-16^c^)**									
	Intercept	–0.8547		0.8088		–1.06 (336.2)		.29	
	Treatment	0.5978		1.0520		0.57 (247.3)		.57	
	Parent gender	1.0437		0.6333		1.65 (244.8)		.101	
	Linear time	–2.3895		0.5498		–4.35 (887.7)		<.001	
	Quadratic time	0.1802		0.5564		0.32 (874.1)		.75	
	Parent gender×linear time	–1.3776		0.6293		–2.19 (890.0)		.03	
	Parent gender×quadratic time	0.2033		0.6445		0.32 (880.1)		.75	
	Treatment×linear time	1.3444		0.6323		2.13 (880.7)		.03	
	Treatment×quadratic time	–0.4098		0.6465		–0.63 (873.4)		.53	
**Social support (Short MOS-SSS^d^)**									
	Intercept	–0.5233		0.2177		–2.40 (372.9)		.02	
	Treatment	0.3073		0.2681		1.15 (235.3)		.25	
	Parent gender	0.7202		0.2135		3.37 (243.9)		<.001	
	Linear time	–0.2292		0.2044		–1.12 (882.1)		.26	
	Quadratic time	0.17140		0.2072		0.83 (865.8)		.41	
	Parent gender×linear time	–0.1775		0.2344		–0.76 (880.1)		.45	
	Parent gender×quadratic time	–0.1654		0.2402		–0.69 (870.6)		.49	
	Treatment×linear time	0.1808		0.2350		0.77 (876.9)		.44	
	Treatment×quadratic time	–0.0543		0.2407		–0.23 (867.7)		.82	
**Parenting self-efficacy^e^**									
	Intercept	–0.9291		1.1960		–0.78 (472.2)		.44	
	Treatment	–0.2268		1.6960		–0.13 (477.2)		.89	
	Parent gender	3.2155		1.6180		1.99 (238.7)		.048	
	Linear time	11.3882		1.3400		8.50 (889.7)		<.001	
	Quadratic time	–1.3224		1.3535		–0.98 (868.3)		.33	
	Parent gender×Treatment	0.0261		2.2939		0.01 (240.8)		.99	
	Parent gender×linear time	1.9064		1.8762		1.02 (883.0)		.31	
	Parent gender×quadratic time	0.1665		1.9024		0.09 (867.1)		.93	
	Treatment×linear time	–3.3766		1.8945		–1.78 (889.1)		.08	
	Treatment×quadratic time	–1.7702		1.9429		–0.91 (878.4)		.36	
	Treatment×parent gender×linear time	6.8500		2.6569		2.58 (883.5)		.01	
	Treatment×parent gender×quadratic time	2.9442		2.7200		1.08 (874.5)		.28	
**Self-efficacy for support provision**									
	Intercept	3.8583		1.1927		3.24 (418.2)		.001	
	Treatment	–0.7276		1.4183		–0.51 (245.0)		.61	
	Parent gender	–6.7124		1.2864		–5.22 (241.3)		<.001	
	Linear time	–0.5999		1.1922		–0.50 (893.7)		.62	
	Quadratic time	1.4951		1.2085		1.24 (877.1)		.22	
	Parent gender×linear time	3.6372		1.3681		2.66 (890.8)		.008	
	Parent gender×quadratic time	–0.2716		1.3996		–0.19 (880.9)		.85	
	Treatment×linear time	3.5459		1.3695		2.59 (888.7)		.01	
	Treatment×quadratic time	–2.2494		1.4006		–1.61 (879.4)		.11	

^a^EPDS: Edinburgh Postnatal Depression Scale.

^b^AQoL-8D: Assessment of Quality of Life-8 Dimensions.

^c^CSI-16: Couples Satisfaction Index-16.

^d^MOS-SSS: Medical Outcomes Study Social Support Survey.

^e^The reported effects are from the hypothesized model, except for parenting self-efficacy, which is from the full factorial model.

### Secondary Outcomes

Relationship satisfaction on the CSI-16 fell over the course of the study (Cohen *d*=–0.36), especially for the mothers (Cohen *d*=–0.46 vs –0.26 for the fathers). However, the reduction in relationship satisfaction was less in *Baby Steps Wellbeing* than in *Baby Care* (Cohen *d*=0.20). Significant interactions with treatment were also observed in the two self-efficacy measures. In the case of self-efficacy for support provision, the *Baby Steps Wellbeing* participants showed a greater linear increase over time than those in *Baby Care* (Cohen *d*=0.26). Across the whole study, the mothers had lower scores than the fathers (Cohen *d*=–0.33), but their scores improved to a greater extent over the study period (Cohen *d*=0.34 vs 0.08 for the fathers). The interaction of time and parent gender was not further modified by treatment. In contrast, the mothers had higher scores on parenting self-efficacy than the fathers on average (Cohen *d*=0.17), as well as showing a greater linear improvement over time (Cohen *d*=1.12 vs 0.72 for the fathers). In this case, there was also a three-way interaction: as presented in Figure 2, the mothers tended to have greater increases in *Baby Steps Wellbeing* than in *Baby Care* (Cohen *d*=0.26), but the reverse was the case for the fathers (Cohen *d*=–0.25). The only secondary outcome not showing any interactions between treatment and time was social support on the Short MOS-SSS. The mothers scored somewhat more highly than the fathers across the study period (Cohen *d*=0.22), but there were no other significant effects.

## Discussion

### Principal Findings

Although satisfaction with the *Baby Care* and *Baby Steps Wellbeing* programs was moderately high, the average number of log-ins and viewed modules was suboptimal, especially for the fathers. Joint access to the program by some partners may have led to underestimates of individual access, but little use was made of the key planning and goal-setting segments of *Baby Steps Wellbeing*.

Consistent with this limited access, perinatal distress on the EPDS, psychosocial quality of life, and social support on the Short MOS-SSS did not show significant differential improvements for the two treatments. However, there was some evidence of differential benefits from the full, interactive *Baby Steps Wellbeing* program compared with the *Baby Care* information modules in 3 of the 4 secondary outcomes. The participants who received the full program had a greater linear rise in self-efficacy for support provision and an attenuated reduction in relationship satisfaction over the study than those who only received *Baby Care* information. Furthermore, consistent with greater program access by the mothers, they had greater linear increases than the fathers in their parenting self-efficacy if they received *Baby Steps Wellbeing* than if they were only given *Baby Care* information*.* These results are partially consistent with those from a recent universal prevention trial on parental stress, which used a booklet, video, home visit, and telephone call focused on baby care and being sensitive to the needs of both the parents and the baby and did not detect benefits over usual care on the EPDS [32]. However, that earlier study did not find differential effects on any other outcomes, including a single-item measure of parenting self-efficacy.

The interpretation of greater benefits from the full *Baby Steps Wellbeing* program is moderated by small effect sizes and by the fact that self-efficacy for support provision comprised a new single-item measure. In addition, the results from analyses in Multimedia Appendix 1, Table S3 that used a nonpreferred model differ from those in the preferred-model analyses, casting doubt on the robustness of the reported effects, and post hoc analyses showed that the significant interactions between time and treatment for 2 outcomes (relationship satisfaction and parenting self-efficacy) were insufficient to give a better model fit compared with a model that only included parent gender and time. Furthermore, all significant interactions between treatment and time involved linear changes to 6 months, rather than specifically modifying outcomes immediately after childbirth, when the greatest vulnerability is seen.

However, the fact that some significant effects were seen at all remained noteworthy, given the limited degree of program involvement and the fact that this was an unselected sample, with average scores on all outcome variables showing little dysfunction throughout the study. In this context, the lack of significant differential effects of the treatments on EPDS scores should not have been surprising, given the study’s low point-prevalence of screening positive for self-reported depression (3% of the fathers and 9% of the mothers at 3 months, using an EPDS cutoff at 12/13) compared with the rates in meta-analyses (8% of fathers [3] and 12%-17% of mothers [1,2]), which restricted the opportunity to detect differential changes.

There were insufficient couples who both accessed and set goals for multiple *Baby Steps* *Wellbeing* modules or continued to use the program postnatally (ie, for more than approximately 10 weeks) to allow a post hoc examination of whether more sustained or intensive program use would have provided stronger results, but this study’s results clearly suggest that these should be goals for future research. Our program had already attempted to maximize ongoing program engagement in multiple ways: (1) by recruiting couples who might reinforce each other’s engagement in program use and resultant actions; (2) by providing information related to baby care, which we expected to have salience late in the pregnancy; (3) by repeatedly reminding participants to log in and giving brief tips that changed at each visit; and (4) by offering opportunities to upload photos that would cue memories of good times with their baby and make it pleasurable to visit the site. We timed entry into the study and initial program exposure while the women were pregnant because of the time pressures experienced after childbirth, but solutions to childcare or general well-being issues might not have immediately seemed relevant to an unselected sample at that time. Furthermore, recruitment primarily through prenatal classes could have resulted in redundancy in some baby care information. In fact, a median of 1-2 website visits suggested that many participants may have regarded the program as just one of many web-based information repositories on baby care, which they only used once.

The absence of a predetermined, sequential progression through the program’s modules might have been a significant factor in the limited use of the program [33,34]. Gamification of progression may increase program use (eg, rewarding module completion or progressive gains in knowledge or skills and inclusion of animations or videos or audios that modeled functional problem solutions [35,36]). The positive effects of coaching on maintained program engagement [17] could be simulated by digital *coaches* who might offer some features that approximated human interaction (eg, greeting and praising completion of tasks) [37], even if the individualization of these responses was limited. Although motivational interviewing before beginning the program may be more feasible in a depression treatment program than in a program that seeks to prevent depression [35], a brief digital adaptation of motivational interviewing (together with a rationale for regular program use) may also assist, especially in relation to addressing the couple’s well-being. Impact and perceived relevance of the program might also be increased if the SMS text messages incorporated tips [38] or empowering messages rather than just reminding users to return to the program.

Problems with engaging fathers in prevention or treatment of mental health conditions are endemic, both perinatally and more generally [8,9], and occur for digital interventions as well as face-to-face services [19]. We had hoped that the provision of a module especially for fathers would increase its perceived relevance and that parallel use of the program by mothers would encourage its use by fathers. However, it seems that further refinement of the approach with a more extensive co-design is required.

There were several indications that the mothers reported more pronounced mental health and well-being issues than the fathers in this study, particularly in the perinatal period. They had higher average EPDS scores as well as lower psychosocial quality of life and self-efficacy for support provision over the course of the study, together with a greater tendency for their EPDS scores to peak. Their quality of life rose less strongly soon after childbirth, and their relationship satisfaction fell over time. Although they had slightly higher average social support over the course of the study and (not unexpectedly) were more confident about their parenting skills than the fathers, these gender differences further substantiate the particular vulnerability of new mothers to issues with mental health and well-being [1-3].

### Strengths and Limitations

To our knowledge, this is the first controlled trial that specifically attempted to engage fathers in a digital perinatal intervention and provided an interactive module especially for them. The trial had a substantial sample size and a very high retention in postbaseline assessments. Randomization was performed through an automated trial management program that ensured equivalence between treatment groups of the EPDS scores at baseline. All assessments were conducted on the web, and the follow-up research staff were blinded to the participants’ allocation. We conducted intention-to-treat analyses, with secondary analyses examining effects in the participants who accessed the program. However, the study’s predominant recruitment through prenatal classes and the sample’s high rate of university education mean that the results may not be generalizable to populations that are less highly educated or less engaged in learning about birthing and childcare. Although recruitment during the third trimester was intended to maximize the perceived relevance of the content, this may also have reduced its perceived novelty as well as excluding parents with early births or pregnancy complications from the sample. The exclusion of parents who were not cohabiting, were not in a male-female relationship, were aged below 18 years, or were expecting multiple infants also restricted the potential application of this study.

### Conclusions

There was some evidence of differential benefits from the full *Baby Steps Wellbeing* program, although these benefits were more consistently seen in the mothers than in the fathers, and the effect sizes were relatively small. A major contributor to these results may have been the low level of use of the self-guided program, especially by the fathers. Increasing this program’s use will be a prerequisite for more accurate future estimation of the degree of potential impact of the *Baby Steps Wellbeing* program on the well-being of new parents.

## Appendix

Multimedia Appendix 1 Supplementary tables.

Multimedia Appendix 2 CONSORT-eHEALTH checklist (V 1.6.1).

## Abbreviations

AQoL-8D: Assessment of Quality of Life-8 Dimensions
CONSORT: Consolidated Standards of Reporting Trials
CSI-16: Couples Satisfaction Index-16
EPDS: Edinburgh Postnatal Depression Scale
MOS-SSS: Medical Outcomes Study Social Support Survey
