# Supplementary material for: The Baby Steps Web Program for the Well-Being of New Parents: Randomized Controlled Trial
Source: J Med Internet Res. 2021 Nov 26;23(11):e23659. doi: 10.2196/23659 (PMC8665385; doi:10.2196/23659)
Supplement: Multimedia Appendix 1 [file jmir_v23i11e23659_app1.docx]

**Multimedia Appendix 1. Supplementary Tables.**

**Table S1. Participants screening positive on the EPDS, using different criteria**

|  | Baseline | | 3 Months | | 6 Months | | Screened positive at any time | |
| --- | --- | --- | --- | --- | --- | --- | --- | --- |
|  | Fathers  N  (%) | Mothers  N  (%) | Fathers  N  (%) | Mothers  N  (%) | Fathers  N  (%) | Mothers  N  (%) | Fathers  N  (%) | Mothers  N  (%) |
| Fathers 5/6;  Mothers 7/8)^a^ | 78  (31.5%) | 68  (27.4%) | 69  (33.0%) | 71  (32.7%) | 57  (27.3%) | 56  (23.7%) | 120 (48.4%) | 127 (51.2%) |
| Fathers 9/10;  Mothers 8/9)^b^ | 24  (9.7%) | 49  (19.8%) | 19  (9.1%) | 59  (27.2%) | 12  (5.7%) | 44  (20.2%) | 46  (18.5%) | 103 (41.5%) |
| 11/12 | 15  (6.0%) | 19  (7.7%) | 9  (4.3%) | 29  (13.4%) | 4  (1.9%) | 14  (6.4%) | 27  (10.9%) | 49  (19.8%) |
| 12/13^c^ | 11  (4.4%) | 15  (6.0%) | 6  (2.9%) | 20  (9.1%) | 2  (1.0%) | 10  (4.6%) | 18  (7.3%) | 40  (16.1%) |
| Total | 248 | 248 | 209 | 217 | 209 | 218 | 248 | 248 |

a. Matthey et al. (Major or minor depression) [20] b. Matthey et al. (Major or minor depression) [20]

c. The most common cutoff in Edward et al. [8]

**Table S2. Program Usage and Satisfaction**^a^

|  | Treatment | | | Parent gender | | | Parent gender x treatment | | | Correlation with baseline EPDS | |
| --- | --- | --- | --- | --- | --- | --- | --- | --- | --- | --- | --- |
|  | *t* | *df* | *P* | *t* | *df* | *P* | *t* | *df* | *P* | *r* | *P* |
| Logins | -0.60 | 487.4 | .550 | 4.94 | 246 | <.001 | 0.68 | 246 | .499 | .03 | .529 |
| Duration of use (days) | 0.24 | 488.4 | .809 | 4.31 | 246 | <.001 | 0.70 | 246 | .485 | .01 | .862 |
| Modules viewed |  |  |  |  |  |  |  |  |  |  |  |
| Total | 0.33 | 488.8 | .743 | 5.30 | 246.0 | <.001 | 2.35 | 246.0 | .020 | .05 | .251 |
| Babycare | -3.40 | 492 | <.001 | 7.22 | 492 | <.001 | -0.36 | 492 | .717 | .03 | .563 |
| Program satisfaction^b^ | 0.54 | 397.5 | .592 | 1.07 | 202.8 | .287 | -0.26 | 204.1 | .798 | -.18 | .029 |

a. Analyses include a random intercept for couple.

b. Uses the average of responses to items on overall satisfaction, relevance, usefulness, how it was easy to find what they wanted.

**Table S3. Outcomes, using Non-Preferred Model**

| Dependent variable | Estimate | SE | df | *t* | *p* |
| --- | --- | --- | --- | --- | --- |
| Depression (EPDS) |  |  |  |  |  |
| Intercept | -0.6167 | 0.2774 | 492.5 | -2.22 | .027 |
| Treatment | -0.2976 | 0.3930 | 496.1 | -0.76 | .449 |
| Parent gender | 1.3484 | 0.3755 | 244.2 | 3.59 | <.001 |
| Linear time | -0.5326 | 0.2760 | 899.6 | -1.93 | .054 |
| Quadratic time | 0.0864 | 0.2784 | 879.9 | 0.31 | .756 |
| Parent gender x treatment | 0.2374 | 0.5320 | 245.8 | 0.45 | .656 |
| Parent gender x linear time | 0.8134 | 0.3863 | 894.1 | 2.11 | .036 |
| Parent gender x quadratic time | -0.7482 | 0.3913 | 878.9 | -1.91 | .056 |
| Treatment x linear time | 0.1971 | 0.3896 | 898.3 | 0.51 | .613 |
| Treatment x quadratic time | -0.3519 | 0.3997 | 887.6 | -0.88 | .379 |
| Treatment x Parent gender x linear time | -0.9203 | 0.5467 | 894.2 | -1.68 | .093 |
| Treatment x Parent gender x  quadratic time | 0.1948 | 0.5596 | 884.7 | 0.34 | .728 |
| Quality of Life (AQoL-  8D-Psychosocial)* |  |  |  |  |  |
| Intercept | 0.0174 | 0.0134 | 496.5 | 1.30 | .193 |
| Treatment | 0.0163 | 0.0189 | 466.2 | 0.86 | .389 |
| Parent gender | -0.0506 | 0.0162 | 247.2 | -3.13 | .002 |
| Linear time | 0.0396 | 0.0105 | 888.2 | 3.78 | <.001 |
| Quadratic time | -0.0057 | 0.0106 | 874.3 | -0.54 | .591 |
| Parent gender x treatment | 0.0121 | 0.0229 | 248.1 | 0.53 | .597 |
| Parent gender x linear time | -0.0136 | 0.0147 | 886.2 | -0.93 | .354 |
| Parent gender x quadratic time | 0.0235 | 0.0149 | 874.3 | 1.58 | .114 |
| Treatment x linear time | -0.0266 | 0.0148 | 887.0 | -1.79 | .073 |
| Treatment x quadratic time | 0.0058 | 0.0152 | 878.9 | 0.38 | .702 |
| Treatment x parent gender x linear time | 0.0448 | 0.0208 | 886.3 | 2.16 | .031 |
| Treatment x parent gender x quadratic time | 0.0059 | 0.0213 | 877.9 | 0.28 | .781 |
| Relationship Satisfaction (CSI-16) |  |  |  |  |  |
| Intercept | -0.2440 | 0.8678 | 409.1 | -0.28 | .779 |
| Treatment | -0.6275 | 1.2287 | 411.0 | -0.51 | .610 |
| Parent gender | -0.1673 | 0.8903 | 242.7 | -0.19 | .851 |
| Linear time | -2.2302 | 0.6368 | 888.5 | -3.50 | <.001 |
| Quadratic time | 0.2590 | 0.6411 | 872.0 | 0.40 | .686 |
| Parent gender x treatment | 2.4308 | 1.2619 | 244.4 | 1.93 | .055 |
| Parent gender x linear time | -1.6776 | 0.8895 | 887.3 | -1.89 | .060 |
| Parent gender x quadratic time | 0.0525 | 0.9000 | 873.5 | 0.06 | .954 |
| Treatment x linear time | 1.0265 | 0.9005 | 887.6 | 1.14 | .255 |
| Treatment x quadratic time | -0.5567 | 0.9215 | 877.7 | -0.60 | .546 |
| Treatment x parent gender x linear time | 0.6047 | 1.2594 | 888.5 | 0.48 | .631 |
| Treatment x parent gender x  quadratic time | 0.2884 | 1.2904 | 878.8 | 0.22 | .823 |

*Uses the Hypothesised Model: For the full model, refer to Table 4 in the text. All other analyses use the full factorial model.

**Table S3 (Cont.). Outcomes, using Non-Preferred Model**

| Dependent variable | Estimate | SE | df | *t* | *p* |
| --- | --- | --- | --- | --- | --- |
| Social Support (Short  MOS-SSS) |  |  |  |  |  |
| Intercept | -0.4988 | 0.2428 | 453.8 | -2.05 | .041 |
| Treatment | 0.2589 | 0.3440 | 457.6 | 0.75 | .452 |
| Parent gender | 0.6681 | 0.3015 | 241.3 | 2.22 | .028 |
| Linear time | -0.4127 | 0.2366 | 882.4 | -1.74 | .081 |
| Quadratic time | 0.2231 | 0.2087 | 862.5 | 0.94 | .350 |
| Parent gender x treatment | 0.1023 | 0.4275 | 243.3 | 0.24 | .811 |
| Parent gender x linear time | 0.1842 | 0.3309 | 877.4 | 0.56 | .578 |
| Parent gender x quadratic time | -0.2704 | 0.3353 | 862.1 | -0.81 | .420 |
| Treatment x linear time | 0.5475 | 0.3345 | 881.6 | 1.64 | .102 |
| Treatment x quadratic time | -0.1599 | 0.3428 | 870.7 | -0.47 | .641 |
| Treatment x parent gender x linear time | -0.7246 | 0.4686 | 878.3 | -1.55 | .122 |
| Treatment x parent gender x  quadratic time | 0.2130 | 0.4804 | 869.1 | 0.44 | .658 |
| Self-efficacy-Parenting* |  |  |  |  |  |
| Intercept | -0.9560 | 1.0480 | 408.0 | -0.91 | .362 |
| Treatment | -0.1913 | 1.2400 | 237.1 | -0.15 | .878 |
| Parent gender | 3.2345 | 1.1439 | 242.1 | 2.83 | .005 |
| Linear time | 9.6464 | 1.1609 | 890.1 | 8.31 | <.001 |
| Quadratic time | -2.0593 | 1.1779 | 872.8 | -1.75 | .081 |
| Parent gender x linear time | 5.3165 | 1.3326 | 886.1 | 3.99 | <.001 |
| Parent gender x quadratic time | 1.5983 | 1.3639 | 876.8 | 1.17 | .242 |
| Treatment x linear time | 0.1100 | 1.3338 | 885.2 | 0.08 | .934 |
| Treatment x quadratic time | -0.2463 | 1.3649 | 876.0 | -0.18 | .857 |
| Self-efficacy-Support Provision |  |  |  |  |  |
| Intercept | 4.7034 | 1.3558 | 484.1 | 3.47 | <.001 |
| Treatment | -2.411 | 1.9216 | 488.4 | -1.26 | .210 |
| Parent gender | -8.3707 | 1.8153 | 238.6 | -4.61 | <.001 |
| Linear time | -0.3763 | 1.3798 | 893.7 | 0.27 | .785 |
| Quadratic time | 2.0542 | 1.3923 | 873.5 | 1.48 | .140 |
| Parent gender x treatment | 3.3167 | 2.5727 | 240.3 | 1.29 | .199 |
| Parent gender x linear time | 1.7470 | 1.9314 | 888.2 | 0.91 | .366 |
| Parent gender x quadratic time | -1.3601 | 1.9567 | 872.7 | -0.70 | .487 |
| Treatment x linear time | 1.5946 | 1.9511 | 893.0 | 0.82 | .414 |
| Treatment x quadratic time | -3.3818 | 1.9996 | 882.0 | -1.69 | .091 |
| Treatment x parent gender x linear time | 3.7981 | 2.7351 | 888.7 | 1.39 | .165 |
| Treatment x parent gender x  quadratic time | 2.2076 | 2.7988 | 879.0 | 0.79 | .430 |
|  |  |  |  |  |  |

*Uses the Hypothesised Model: For the full model, refer to Table 4 in the text. All other analyses use the full factorial model.

**Table S4. Post-hoc Comparisons of Model Fit between the Preferred Model and a Model only including Parent Gender and Time**

| Dependent variable and Model^a^ | N Parameters | AIC | BIC | Log Likelihood | Deviance | χ^2^ | df | *p* |
| --- | --- | --- | --- | --- | --- | --- | --- | --- |
| Depression (EPDS) |  |  |  |  |  |  |  |  |
| Hypothesised Model | 12 | 7247.7 | 7310.2 | -3611.9 | 7223.7 |  |  |  |
| Parent gender, time | 9 | 7243.8 | 7290.7 | -3612.9 | 7225.8 | 2.12 | 3 | .549 |
| Quality of Life (AQoL-8D Psychosocial) |  |  |  |  |  |  |  |  |
| Hypothesised Model | 12 | -1361.4 | -1298.9 | 692.7 | -1385.4 |  |  |  |
| Parent gender, time | 9 | -1364.1 | -1317.2 | 691.1 | -1382.1 | 3.26 | 3 | .353 |
| Relationship Satisfaction (CSI-16) |  |  |  |  |  |  |  |  |
| Hypothesised Model | 12 | 9716.4 | 9778.9 | -4846.2 | 9692.4 |  |  |  |
| Parent gender, time | 9 | 9715.7 | 9762.5 | -4848.8 | 9607.7 | 5.26 | 3 | .154 |
| Social Support (Short MOS-SSS) |  |  |  |  |  |  |  |  |
| Hypothesised Model | 12 | 6826.2 | 6888.7 | -3401.1 | 6802.2 |  |  |  |
| Parent gender, time | 9 | 6822.1 | 6868.9 | -3402.0 | 6804.1 | 1.88 | 3 | .598 |
| Parenting Self-Efficacy |  |  |  |  |  |  |  |  |
| Full model | 15 | 11394 | 11472 | -5681.9 | 11364 |  |  |  |
| Parent gender, time | 9 | 11389 | 11436 | -5685.7 | 11371 | 7.72 | 6 | .260 |
| Self-Efficacy for Support Provision |  |  |  |  |  |  |  |  |
| Hypothesised Model | 12 | 11560 | 11622 | -5768.0 | 11536 |  |  |  |
| Parent gender, time | 9 | 11564 | 11611 | -5773.0 | 11546 | 14.16 | 3 | .028 |

1. All models have random intercepts for subject and couple. Full model: full factorial design. Hypothesised Model: Treatment, parent gender, time, treatment x time, gender x time. Parent gender and time only: parent gender, time, parent gender x time.

**Table S5. Predicted Means for Outcome Variables from Baseline to 6 Months, using Preferred Model**

| Outcomes | Babycare Only | | | Baby Steps Wellbeing | | |
| --- | --- | --- | --- | --- | --- | --- |
|  | Baseline  M (SE) | 3 Months  M (SE) | 6 Months  M (SE) | Baseline  M (SE) | 3 Months  M (SE) | 6 Months  M (SE) |
| Primary |  |  |  |  |  |  |
| Depression (EPDS total) |  |  |  |  |  |  |
| Fathers | 4.52 | 4.26 | 4.09 | 4.42 | 4.28 | 3.62 |
|  | (0.30) | (0.32) | (0.32) | (0.30) | (0.32) | (0.32) |
| Mothers | 5.47 | 6.26 | 5.54) | 5.37 | 6.28 | 5.07 |
|  | (0.30) | (0.32) | (0.32) | (0.30) | (0.32) | (0.32) |
| Quality of Life (AQoL-8D Psychosocial)* |  |  |  |  |  |  |
| Fathers | 0.455 | 0.490 | 0.511 | 0.493 | 0.502 | 0.511 |
|  | (0.015) | (0.016) | (0.016) | (0.015) | (0.016) | (0.016) |
| Mothers | 0.424 | 0.421 | 0.461 | 0.444 | 0.439 | 0.507 |
|  | (0.015) | (0.016) | (0.016) | (0.015) | (0.016) | (0.016) |
| Secondary |  |  |  |  |  |  |
| Relationship satisfaction (CSI-16) |  |  |  |  |  |  |
| Fathers | 70.1 | 68.2 | 66.7 | 69.6 | 69.1 | 68.1 |
|  | (0.90) | (0.94) | (0.94) | (0.90) | (0.95) | (0.94) |
| Mothers | 72.2 | 69.1 | 66.9 | 71.7 | 70.0 | 68.3 |
|  | (0.90) | (0.93) | (0.93) | (0.90) | (0.94) | (0.94) |
| Social Support (Short MOS-SSS) |  |  |  |  |  |  |
| Fathers | 15.8 | 15.4 | 15.4 | 15.9 | 15.7 | 15.9 |
|  | (0.264) | (0.279) | (0.281) | (0.264) | (0.284) | (0.282) |
| Mothers | 16.5 | 16.3 | 16.0 | 16.7 | 16.6 | 16.4 |
|  | (0.264) | (0.278) | (0.278) | (0.264) | (0.281) | (0.279) |
| Self-efficacy for parenting* |  |  |  |  |  |  |
| Fathers | 64.5 | 74.2 | 80.6 | 65.9 | 75.4 | 77.3 |
|  | (1.55) | (1.65) | (1.67) | (1.55) | (1.69) | (1.67) |
| Mothers | 66.4 | 77.2 | 85.2 | 64.2 | 76.1 | 88.0 |
|  | (1.55) | (1.63) | (1.63) | (1.55) | (1.65) | (1.65) |
| Self-efficacy for Support Provision |  |  |  |  |  |  |
| Fathers | 83.2 | 81.0 | 82.4 | 79.1 | 82.1 | 83.3 |
|  | (1.48) | (1.57) | (1.58) | (1.48) | (1.59) | (1.58) |
| Mothers | 73.9 | 74.5 | 78.1 | 69.7 | 75.6 | 79.0 |
|  | (1.48) | (1.56) | (1.56) | (1.48) | (1.57) | (1.57) |

*Uses the full factorial model to calculate predicted means and standard errors. All others use the Hypothesised Model.
